# Bis(dicyclo­hexyl­phenyl­phosphine)iodido­silver(I) pyridine monosolvate

**DOI:** 10.1107/S1600536809040732

**Published:** 2009-10-10

**Authors:** Bernard Omondi, Reinout Meijboom

**Affiliations:** a Department of Chemistry, University of Johannesburg, PO Box 524, Auckland Park, Johannesburg 2006, South Africa

## Abstract

The structure of the title compound, [AgI(C_18_H_27_P)_2_]·C_5_H_5_N, shows a trigonal-planar coordinated Ag^I^ atom within a distorted IAgP_2_ donor set. The pyridine solvent mol­ecule is only associated with the complex *via* very weak inter­molecular C—H⋯N inter­actions.

## Related literature

For general background to silver(I) phosphine complexes, see: Meijboom *et al.* (2009 ▶). For related structures, see: Bowmaker *et al.* (1993 ▶, 1996 ▶); Alyea *et al.* (1982 ▶); Lin *et al.* (1993 ▶). For the solution behaviour of [Ag*XL*_n_] complexes (*L* = tertiary phosphine, *n* = 1–4, *X* = coordinating or non-coordinating anion), see: Muetterties & Alegranti (1972 ▶).
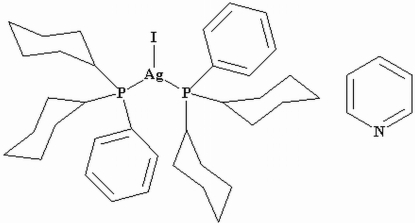


## Experimental

### 

#### Crystal data

[AgI(C_18_H_27_P)_2_]·C_5_H_5_N
*M* *_r_* = 862.13
Monoclinic, 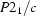

*a* = 18.696 (4) Å
*b* = 11.874 (2) Å
*c* = 23.641 (8) Å
β = 128.131 (18)°
*V* = 4128 (2) Å^3^
*Z* = 4
Mo *K*α radiation
μ = 1.34 mm^−1^
*T* = 298 K
0.34 × 0.20 × 0.16 mm

#### Data collection

Bruker APEXII CCD area-detector diffractometer
Absorption correction: multi-scan (*SADABS*; Bruker, 2004 ▶) *T*_min_ = 0.659, *T*_max_ = 0.814
27061 measured reflections
10220 independent reflections
6255 reflections with *I* > 2σ(*I*)
*R*_int_ = 0.041

#### Refinement

*R*[*F* ^2^ > 2σ(*F* ^2^)] = 0.035
*wR*(*F* ^2^) = 0.079
*S* = 0.99
10220 reflections
415 parameters
H-atom parameters constrained
Δρ_max_ = 0.54 e Å^−3^
Δρ_min_ = −0.59 e Å^−3^

### 

Data collection: *APEX2* (Bruker, 2005 ▶); cell refinement: *SAINT-Plus* (Bruker, 2004 ▶); data reduction: *SAINT-Plus* and *XPREP* (Bruker, 2004 ▶); program(s) used to solve structure: *SHELXS97* (Sheldrick, 2008 ▶); program(s) used to refine structure: *SHELXL97* (Sheldrick, 2008 ▶); molecular graphics: *ORTEP-3* (Farrugia, 1997 ▶); software used to prepare material for publication: *WinGX* (Farrugia, 1999 ▶).

## Supplementary Material

Crystal structure: contains datablocks global, I. DOI: 10.1107/S1600536809040732/hg2571sup1.cif

Structure factors: contains datablocks I. DOI: 10.1107/S1600536809040732/hg2571Isup2.hkl

Additional supplementary materials:  crystallographic information; 3D view; checkCIF report

## Figures and Tables

**Table:** 

I—Ag	2.7725 (5)
Ag—P2	2.4462 (9)
Ag—P1	2.4643 (9)

**Table:** 

P2—Ag—P1	131.59 (3)
P2—Ag—I	122.75 (2)
P1—Ag—I	105.00 (2)

**Table 2:** Hydrogen-bond geometry (Å, °)

*D*—H⋯*A*	*D*—H	H⋯*A*	*D*⋯*A*	*D*—H⋯*A*
C66—H66⋯N^i^	0.93	2.72	3.538 (4)	147

Symmetry code: (i) 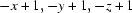.

**Fig. 1.**
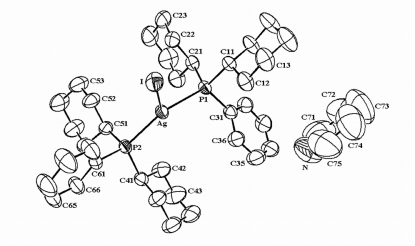
The molecular structure of (I), showing 50% probability displacement ellipsoids. H atoms have been omitted for clarity.

## supplementary crystallographic information

### Comment

Stoichiometric reactions of silver(I) with tertiary phosphines often results in silver(I) complexes of the type [AgXL_n_] (L = tertiary phosphine; n = 1 - 4 ; X = coordinating or non-coordinating anion). These complexes display a diversity of structural types, and reviews on this topic have been published (Meijboom *et al.*, 2009 and refs. therein). A 1:2 stoichiometric ratio generally results in monomeric complex [AgX(PR_3_)_2_]/[Ag(PR_3_)_2_]^+^X^-^ or dimeric complex [{AgXL_2_}_2_] (Bowmaker *et al.*, 1996; Meijboom *et al.*,  2009) depending on the donor properties of the phosphine ligand, the bulkiness of the ligand substituents and the donor properties of the anion (Bowmaker *et al.*, 1996).

The title complex crystallizes as mononuclear units in the P2_1_/n space group with one [AgBr{PCy_2_Ph}_2_] complex and one pyridine molecule in the asymetric unit as expected for the bulky and fairly basic dicyclohexylphenyl phosphine ligands (Lin *et al.*, 1993; Alyea *et al.*,  1982; Bowmaker *et al.*,  1993). This type of [AgX(PR_3_)_2_] coordination was also observed for X = CN, I, Br, C1, SCN or NCO, most of which were found to be isomorphous in the monoclinic C2/c space group (Bowmaker *et al.*,  1996).

The iodide anion is unsymmetrically coordinated to the silver with I-Ag-P angles of 105.00 (2) and 122.75 (2)°. The P-Ag-P angle is 131.59 (3)° with the I-Ag distance being 2.7725 (5) Å. These angles and distances are comparable to those of the thiocyanate analogue ([AgSCN(P{Cy_3_})_2_] I-Ag-P = 104.60 (8) and 123.69 (8)° and P-Ag-P = 131.51 (7)°) (Bowmaker *et al.*,  1996) both of which have the disposition of the two phosphine ligands fairly different. This fits with trend that relates M-X distances and P-M-P angles as shown by Bowmaker *et al.* (1996) for complexes with bulky phosphines. The three-co-ordinate (P_2_AgX) silver environment is planar with the sum of the I-Ag-P and P-Ag-P angles being 359.3°. The pyridine solvate interacts very weakly with the silver(I) complex through C-H···N interactions.

Despite the number of structural reports of [AgXL_n_] complexes, their solution behaviour, initiated by Muetterties & Alegranti (1972), has always shown that the coordinating ligands were labile in all complexes studied. Rapid ligand-exchange reactions have been reported for all ^31^P NMR spectroscopic investigations of ionic Ag^I^ monodentate phosphine complexes, thus making NMR spectroscopy of limited use for these types of complexes.

### Experimental

Silver iodide (0.130 g, 0.43 mmol) and dicyclohexylphenylphosphine (1.009 g, 0.86 mmol) were suspended in pyridine (5 ml). The mixture was heated to give a clear solution. Colourless crystals of the title compound suitable for X-ray crystallography were obtained by slow evaporation.

### Refinement

All hydrogen atoms were positioned geometrically, with C–H = 0.97 Å, and allowed to ride on their parent atoms with U_iso_(H) = 1.2U_eq_(C).

### Crystal data

**Table:**

[AgI(C_18_H_27_P)_2_]·C_5_H_5_N	*F*(000) = 1768
*M**_r_* = 862.13	*D*_x_ = 1.387 Mg m^−^^3^
Monoclinic, *P*2_1_/*c*	Mo *K*α radiation, λ = 0.71073 Å
Hall symbol: -P 2ybc	Cell parameters from 27740 reflections
*a* = 18.696 (4) Å	θ = 1.4–28.3°
*b* = 11.874 (2) Å	µ = 1.34 mm^−^^1^
*c* = 23.641 (8) Å	*T* = 298 K
β = 128.131 (18)°	Cuboid, colourless
*V* = 4128 (2) Å^3^	0.34 × 0.20 × 0.16 mm
*Z* = 4	

### Data collection

**Table:**

Bruker APEXII CCD area-detector diffractometer	6255 reflections with *I* > 2σ(*I*)
Detector resolution: 0 pixels mm^-1^	*R*_int_ = 0.041
ω scans	θ_max_ = 28.3°, θ_min_ = 1.4°
Absorption correction: multi-scan (*SADABS*; Bruker, 2004)	*h* = −24→23
*T*_min_ = 0.659, *T*_max_ = 0.814	*k* = −15→15
27061 measured reflections	*l* = −31→27
10220 independent reflections	

### Refinement

**Table:**

Refinement on *F*^2^	0 restraints
Least-squares matrix: full	H-atom parameters constrained
*R*[*F*^2^ > 2σ(*F*^2^)] = 0.035	*w* = 1/[σ^2^(*F*_o_^2^) + (0.0275*P*)^2^ + 0.9189*P*] where *P* = (*F*_o_^2^ + 2*F*_c_^2^)/3
*wR*(*F*^2^) = 0.079	(Δ/σ)_max_ = 0.005
*S* = 0.99	Δρ_max_ = 0.54 e Å^−^^3^
10220 reflections	Δρ_min_ = −0.59 e Å^−^^3^
415 parameters	

### Special details

**Table:**

Geometry. All esds (except the esd in the dihedral angle between two l.s. planes) are estimated using the full covariance matrix. The cell esds are taken into account individually in the estimation of esds in distances, angles and torsion angles; correlations between esds in cell parameters are only used when they are defined by crystal symmetry. An approximate (isotropic) treatment of cell esds is used for estimating esds involving l.s. planes.

### Fractional atomic coordinates and isotropic or equivalent isotropic displacement parameters (Å^2^)

**Table:**

	*x*	*y*	*z*	*U*_iso_*/*U*_eq_	
I	0.790785 (18)	0.204293 (18)	0.615050 (14)	0.05957 (8)	
Ag	0.744504 (16)	0.379062 (18)	0.664695 (13)	0.03984 (7)	
P1	0.85008 (5)	0.53299 (6)	0.69227 (4)	0.03805 (19)	
P2	0.63620 (6)	0.35663 (6)	0.68953 (5)	0.03993 (19)	
C11	0.9128 (2)	0.5133 (3)	0.65564 (16)	0.0422 (8)	
H11	0.941	0.4388	0.6722	0.051*	
C12	0.8498 (2)	0.5074 (3)	0.57406 (18)	0.0578 (10)	
H12A	0.8043	0.4497	0.5582	0.069*	
H12B	0.8187	0.5789	0.5545	0.069*	
C13	0.9017 (3)	0.4810 (3)	0.5454 (2)	0.0721 (12)	
H13A	0.8604	0.485	0.4933	0.086*	
H13B	0.9251	0.4047	0.5589	0.086*	
C14	0.9798 (3)	0.5618 (4)	0.5742 (2)	0.0878 (14)	
H14A	1.0146	0.5377	0.5585	0.105*	
H14B	0.9558	0.6362	0.5546	0.105*	
C15	1.0410 (3)	0.5679 (4)	0.6541 (2)	0.0831 (14)	
H15A	1.0867	0.6254	0.6699	0.1*	
H15B	1.0722	0.4964	0.6736	0.1*	
C16	0.9900 (2)	0.5947 (3)	0.68342 (19)	0.0585 (10)	
H16A	1.0317	0.5914	0.7355	0.07*	
H16B	0.966	0.6707	0.6696	0.07*	
C21	0.9404 (2)	0.5455 (3)	0.78958 (16)	0.0440 (8)	
H21	0.9831	0.604	0.7981	0.053*	
C22	0.9919 (2)	0.4344 (3)	0.81973 (18)	0.0557 (9)	
H22A	0.9501	0.3756	0.811	0.067*	
H22B	1.0161	0.4139	0.7948	0.067*	
C23	1.0696 (3)	0.4419 (3)	0.90018 (19)	0.0693 (11)	
H23A	1.1148	0.495	0.9087	0.083*	
H23B	1.0984	0.3688	0.9179	0.083*	
C24	1.0341 (3)	0.4802 (4)	0.9408 (2)	0.0859 (14)	
H24A	0.9953	0.4221	0.9376	0.103*	
H24B	1.085	0.4912	0.9911	0.103*	
C25	0.9813 (3)	0.5880 (4)	0.9101 (2)	0.0898 (15)	
H25A	1.0218	0.6479	0.9179	0.108*	
H25B	0.9572	0.6081	0.9352	0.108*	
C26	0.9030 (3)	0.5781 (3)	0.8298 (2)	0.0656 (11)	
H26A	0.8711	0.6494	0.8117	0.079*	
H26B	0.8604	0.5213	0.8218	0.079*	
C31	0.7997 (2)	0.6729 (3)	0.66097 (18)	0.0446 (8)	
C32	0.8443 (3)	0.7733 (3)	0.69474 (19)	0.0554 (9)	
H32	0.9036	0.7718	0.7374	0.066*	
C33	0.8012 (3)	0.8755 (3)	0.6654 (2)	0.0654 (10)	
H33	0.8297	0.9418	0.6904	0.079*	
C34	0.7164 (3)	0.8799 (3)	0.5992 (2)	0.0753 (12)	
H34	0.6902	0.9488	0.5774	0.09*	
C35	0.6714 (3)	0.7822 (3)	0.5662 (2)	0.0788 (13)	
H35	0.6123	0.7846	0.5234	0.095*	
C36	0.7129 (2)	0.6792 (3)	0.59573 (19)	0.0587 (10)	
H36	0.6821	0.6133	0.5715	0.07*	
C41	0.5531 (2)	0.4716 (3)	0.65743 (17)	0.0446 (8)	
H41	0.5143	0.4543	0.671	0.054*	
C42	0.6003 (3)	0.5839 (3)	0.6913 (2)	0.0652 (10)	
H42A	0.6434	0.5988	0.6822	0.078*	
H42B	0.6336	0.5801	0.7429	0.078*	
C43	0.5308 (3)	0.6802 (3)	0.6603 (3)	0.0932 (16)	
H43A	0.4921	0.6695	0.6742	0.112*	
H43B	0.5628	0.7511	0.6805	0.112*	
C44	0.4724 (3)	0.6859 (3)	0.5792 (3)	0.0831 (14)	
H44A	0.4279	0.7456	0.5616	0.1*	
H44B	0.5102	0.7034	0.5652	0.1*	
C45	0.4247 (3)	0.5765 (3)	0.5463 (2)	0.0729 (11)	
H45A	0.3899	0.5806	0.4945	0.088*	
H45B	0.3828	0.5623	0.5566	0.088*	
C46	0.4936 (2)	0.4795 (3)	0.57595 (18)	0.0608 (10)	
H46A	0.461	0.4091	0.5552	0.073*	
H46B	0.5318	0.4906	0.5617	0.073*	
C51	0.6979 (2)	0.3516 (3)	0.78730 (17)	0.0451 (8)	
H51	0.7319	0.4225	0.806	0.054*	
C52	0.7693 (3)	0.2594 (3)	0.8213 (2)	0.0632 (10)	
H52A	0.7394	0.1868	0.8036	0.076*	
H52B	0.8075	0.2689	0.8069	0.076*	
C53	0.8281 (3)	0.2603 (4)	0.9023 (2)	0.0795 (13)	
H53A	0.8655	0.3276	0.9204	0.095*	
H53B	0.8684	0.1956	0.9211	0.095*	
C54	0.7727 (3)	0.2574 (4)	0.9294 (2)	0.0846 (14)	
H54A	0.7424	0.1851	0.9178	0.102*	
H54B	0.813	0.2657	0.9813	0.102*	
C55	0.7031 (3)	0.3500 (4)	0.8961 (2)	0.0829 (14)	
H55A	0.7339	0.4222	0.9125	0.099*	
H55B	0.6661	0.3432	0.9118	0.099*	
C56	0.6422 (3)	0.3460 (3)	0.8145 (2)	0.0665 (11)	
H56A	0.6	0.4088	0.795	0.08*	
H56B	0.607	0.2769	0.7978	0.08*	
C61	0.5671 (2)	0.2287 (3)	0.65462 (18)	0.0480 (8)	
C62	0.5892 (3)	0.1435 (3)	0.6281 (2)	0.0729 (12)	
H62	0.6385	0.1526	0.6281	0.087*	
C63	0.5392 (3)	0.0449 (3)	0.6015 (3)	0.0965 (16)	
H63	0.5555	−0.0116	0.5841	0.116*	
C64	0.4664 (3)	0.0301 (4)	0.6007 (3)	0.1010 (16)	
H64	0.4328	−0.0362	0.5828	0.121*	
C65	0.4429 (3)	0.1126 (3)	0.6262 (3)	0.0871 (14)	
H65	0.3928	0.1027	0.6252	0.104*	
C66	0.4930 (3)	0.2117 (3)	0.6537 (2)	0.0650 (11)	
H66	0.4767	0.2671	0.6716	0.078*	
N	0.6575 (6)	0.5980 (8)	0.3528 (6)	0.164 (3)	
C71	0.7217 (8)	0.6745 (10)	0.3969 (5)	0.160 (4)	
H71	0.7232	0.7045	0.434	0.192*	
C72	0.7839 (7)	0.7092 (7)	0.3891 (6)	0.185 (4)	
H72	0.8238	0.7678	0.4168	0.222*	
C73	0.7862 (8)	0.6571 (9)	0.3407 (8)	0.191 (5)	
H73	0.8311	0.6758	0.3366	0.229*	
C74	0.7275 (8)	0.5813 (8)	0.2994 (6)	0.170 (4)	
H74	0.7296	0.5454	0.2655	0.204*	
C75	0.6646 (6)	0.5551 (6)	0.3055 (6)	0.163 (3)	
H75	0.6216	0.5018	0.2737	0.196*	

### Atomic displacement parameters (Å^2^)

**Table:**

	*U*^11^	*U*^22^	*U*^33^	*U*^12^	*U*^13^	*U*^23^
I	0.07733 (19)	0.04074 (13)	0.08115 (19)	0.00982 (12)	0.05920 (16)	0.00168 (12)
Ag	0.03998 (14)	0.03724 (13)	0.04897 (15)	−0.00255 (11)	0.03081 (12)	0.00066 (11)
P1	0.0378 (5)	0.0363 (4)	0.0457 (5)	−0.0051 (4)	0.0286 (4)	−0.0027 (4)
P2	0.0401 (5)	0.0362 (4)	0.0511 (5)	0.0002 (4)	0.0320 (4)	0.0025 (4)
C11	0.0417 (19)	0.0467 (18)	0.046 (2)	−0.0037 (15)	0.0309 (17)	−0.0018 (15)
C12	0.061 (2)	0.066 (2)	0.054 (2)	−0.0194 (19)	0.039 (2)	−0.0130 (18)
C13	0.089 (3)	0.083 (3)	0.065 (3)	−0.020 (2)	0.058 (3)	−0.019 (2)
C14	0.101 (4)	0.111 (4)	0.092 (4)	−0.033 (3)	0.080 (3)	−0.028 (3)
C15	0.066 (3)	0.122 (4)	0.086 (3)	−0.035 (3)	0.060 (3)	−0.028 (3)
C16	0.054 (2)	0.074 (2)	0.059 (2)	−0.0286 (19)	0.041 (2)	−0.0205 (19)
C21	0.048 (2)	0.0457 (18)	0.044 (2)	−0.0064 (16)	0.0311 (17)	−0.0043 (15)
C22	0.056 (2)	0.058 (2)	0.053 (2)	−0.0002 (18)	0.034 (2)	0.0043 (17)
C23	0.062 (3)	0.081 (3)	0.050 (3)	0.001 (2)	0.027 (2)	0.015 (2)
C24	0.088 (3)	0.114 (4)	0.049 (3)	−0.009 (3)	0.039 (3)	0.011 (2)
C25	0.113 (4)	0.117 (4)	0.056 (3)	0.003 (3)	0.061 (3)	−0.009 (3)
C26	0.076 (3)	0.080 (3)	0.060 (3)	0.004 (2)	0.052 (2)	−0.003 (2)
C31	0.044 (2)	0.0372 (17)	0.055 (2)	−0.0042 (15)	0.0313 (18)	−0.0020 (15)
C32	0.055 (2)	0.045 (2)	0.063 (2)	−0.0071 (17)	0.035 (2)	−0.0065 (17)
C33	0.074 (3)	0.0356 (19)	0.089 (3)	−0.0066 (19)	0.052 (3)	−0.008 (2)
C34	0.065 (3)	0.045 (2)	0.112 (4)	0.009 (2)	0.053 (3)	0.013 (2)
C35	0.054 (3)	0.059 (3)	0.090 (3)	0.003 (2)	0.028 (2)	0.015 (2)
C36	0.048 (2)	0.0400 (19)	0.068 (3)	−0.0073 (17)	0.026 (2)	−0.0014 (17)
C41	0.0416 (19)	0.0439 (18)	0.053 (2)	0.0007 (15)	0.0314 (17)	0.0009 (15)
C42	0.059 (2)	0.047 (2)	0.069 (3)	0.0040 (18)	0.029 (2)	−0.0089 (18)
C43	0.085 (3)	0.050 (2)	0.106 (4)	0.018 (2)	0.039 (3)	−0.011 (2)
C44	0.075 (3)	0.052 (3)	0.106 (4)	0.024 (2)	0.047 (3)	0.018 (2)
C45	0.060 (3)	0.066 (3)	0.071 (3)	0.018 (2)	0.030 (2)	0.013 (2)
C46	0.059 (2)	0.051 (2)	0.055 (2)	0.0048 (18)	0.026 (2)	0.0022 (17)
C51	0.0440 (19)	0.0502 (19)	0.048 (2)	0.0075 (16)	0.0315 (17)	0.0067 (15)
C52	0.062 (2)	0.079 (3)	0.063 (3)	0.030 (2)	0.045 (2)	0.023 (2)
C53	0.073 (3)	0.103 (3)	0.070 (3)	0.037 (3)	0.048 (3)	0.031 (2)
C54	0.092 (3)	0.113 (4)	0.065 (3)	0.032 (3)	0.057 (3)	0.028 (3)
C55	0.095 (3)	0.110 (3)	0.072 (3)	0.044 (3)	0.066 (3)	0.027 (3)
C56	0.063 (3)	0.086 (3)	0.074 (3)	0.022 (2)	0.054 (2)	0.020 (2)
C61	0.044 (2)	0.0387 (18)	0.066 (2)	−0.0004 (15)	0.0364 (19)	0.0069 (16)
C62	0.076 (3)	0.051 (2)	0.120 (4)	−0.012 (2)	0.075 (3)	−0.016 (2)
C63	0.101 (4)	0.055 (3)	0.165 (5)	−0.021 (3)	0.098 (4)	−0.033 (3)
C64	0.086 (4)	0.054 (3)	0.171 (5)	−0.024 (3)	0.084 (4)	−0.015 (3)
C65	0.073 (3)	0.062 (3)	0.148 (4)	−0.013 (2)	0.079 (3)	0.003 (3)
C66	0.059 (2)	0.053 (2)	0.101 (3)	−0.0053 (19)	0.058 (2)	0.002 (2)
N	0.128 (6)	0.192 (8)	0.225 (9)	0.042 (5)	0.135 (7)	0.050 (6)
C71	0.127 (7)	0.225 (12)	0.124 (7)	0.074 (7)	0.076 (6)	0.025 (6)
C72	0.135 (8)	0.142 (7)	0.252 (12)	−0.001 (6)	0.107 (8)	−0.022 (7)
C73	0.222 (11)	0.113 (7)	0.342 (16)	−0.018 (7)	0.227 (12)	0.022 (8)
C74	0.231 (12)	0.124 (7)	0.254 (10)	0.016 (7)	0.199 (10)	0.018 (7)
C75	0.138 (7)	0.109 (5)	0.252 (11)	0.004 (5)	0.125 (7)	0.002 (6)

### Geometric parameters (Å, °)

**Table:**

I—Ag	2.7725 (5)	C41—C42	1.525 (4)
Ag—P2	2.4462 (9)	C41—H41	0.98
Ag—P1	2.4643 (9)	C42—C43	1.534 (5)
P1—C31	1.825 (3)	C42—H42A	0.97
P1—C21	1.835 (3)	C42—H42B	0.97
P1—C11	1.852 (3)	C43—C44	1.511 (6)
P2—C61	1.828 (3)	C43—H43A	0.97
P2—C51	1.839 (3)	C43—H43B	0.97
P2—C41	1.843 (3)	C44—C45	1.494 (5)
C11—C16	1.509 (4)	C44—H44A	0.97
C11—C12	1.519 (4)	C44—H44B	0.97
C11—H11	0.98	C45—C46	1.536 (5)
C12—C13	1.518 (5)	C45—H45A	0.97
C12—H12A	0.97	C45—H45B	0.97
C12—H12B	0.97	C46—H46A	0.97
C13—C14	1.514 (5)	C46—H46B	0.97
C13—H13A	0.97	C51—C52	1.517 (4)
C13—H13B	0.97	C51—C56	1.528 (4)
C14—C15	1.487 (5)	C51—H51	0.98
C14—H14A	0.97	C52—C53	1.509 (5)
C14—H14B	0.97	C52—H52A	0.97
C15—C16	1.520 (5)	C52—H52B	0.97
C15—H15A	0.97	C53—C54	1.518 (5)
C15—H15B	0.97	C53—H53A	0.97
C16—H16A	0.97	C53—H53B	0.97
C16—H16B	0.97	C54—C55	1.502 (5)
C21—C22	1.526 (4)	C54—H54A	0.97
C21—C26	1.537 (4)	C54—H54B	0.97
C21—H21	0.98	C55—C56	1.518 (5)
C22—C23	1.525 (5)	C55—H55A	0.97
C22—H22A	0.97	C55—H55B	0.97
C22—H22B	0.97	C56—H56A	0.97
C23—C24	1.534 (5)	C56—H56B	0.97
C23—H23A	0.97	C61—C62	1.380 (5)
C23—H23B	0.97	C61—C66	1.387 (4)
C24—C25	1.501 (6)	C62—C63	1.383 (5)
C24—H24A	0.97	C62—H62	0.93
C24—H24B	0.97	C63—C64	1.362 (6)
C25—C26	1.526 (5)	C63—H63	0.93
C25—H25A	0.97	C64—C65	1.356 (6)
C25—H25B	0.97	C64—H64	0.93
C26—H26A	0.97	C65—C66	1.391 (5)
C26—H26B	0.97	C65—H65	0.93
C31—C36	1.389 (5)	C66—H66	0.93
C31—C32	1.390 (4)	N—C75	1.307 (10)
C32—C33	1.383 (5)	N—C71	1.345 (10)
C32—H32	0.93	C71—C72	1.349 (11)
C33—C34	1.379 (5)	C71—H71	0.93
C33—H33	0.93	C72—C73	1.324 (11)
C34—C35	1.361 (5)	C72—H72	0.93
C34—H34	0.93	C73—C74	1.283 (11)
C35—C36	1.383 (5)	C73—H73	0.93
C35—H35	0.93	C74—C75	1.307 (10)
C36—H36	0.93	C74—H74	0.93
C41—C46	1.520 (4)	C75—H75	0.93
			
P2—Ag—P1	131.59 (3)	C46—C41—P2	109.8 (2)
P2—Ag—I	122.75 (2)	C42—C41—P2	111.4 (2)
P1—Ag—I	105.00 (2)	C46—C41—H41	108.5
C31—P1—C21	106.21 (15)	C42—C41—H41	108.5
C31—P1—C11	103.99 (14)	P2—C41—H41	108.5
C21—P1—C11	103.79 (14)	C41—C42—C43	110.9 (3)
C31—P1—Ag	116.06 (11)	C41—C42—H42A	109.5
C21—P1—Ag	111.01 (10)	C43—C42—H42A	109.5
C11—P1—Ag	114.67 (10)	C41—C42—H42B	109.5
C61—P2—C51	105.10 (15)	C43—C42—H42B	109.5
C61—P2—C41	104.42 (15)	H42A—C42—H42B	108
C51—P2—C41	104.89 (14)	C44—C43—C42	112.0 (3)
C61—P2—Ag	116.17 (11)	C44—C43—H43A	109.2
C51—P2—Ag	109.57 (11)	C42—C43—H43A	109.2
C41—P2—Ag	115.63 (10)	C44—C43—H43B	109.2
C16—C11—C12	111.4 (3)	C42—C43—H43B	109.2
C16—C11—P1	115.3 (2)	H43A—C43—H43B	107.9
C12—C11—P1	112.5 (2)	C45—C44—C43	110.7 (3)
C16—C11—H11	105.6	C45—C44—H44A	109.5
C12—C11—H11	105.6	C43—C44—H44A	109.5
P1—C11—H11	105.6	C45—C44—H44B	109.5
C13—C12—C11	111.6 (3)	C43—C44—H44B	109.5
C13—C12—H12A	109.3	H44A—C44—H44B	108.1
C11—C12—H12A	109.3	C44—C45—C46	110.6 (3)
C13—C12—H12B	109.3	C44—C45—H45A	109.5
C11—C12—H12B	109.3	C46—C45—H45A	109.5
H12A—C12—H12B	108	C44—C45—H45B	109.5
C12—C13—C14	111.9 (3)	C46—C45—H45B	109.5
C12—C13—H13A	109.2	H45A—C45—H45B	108.1
C14—C13—H13A	109.2	C41—C46—C45	112.1 (3)
C12—C13—H13B	109.2	C41—C46—H46A	109.2
C14—C13—H13B	109.2	C45—C46—H46A	109.2
H13A—C13—H13B	107.9	C41—C46—H46B	109.2
C15—C14—C13	112.0 (3)	C45—C46—H46B	109.2
C15—C14—H14A	109.2	H46A—C46—H46B	107.9
C13—C14—H14A	109.2	C52—C51—C56	110.6 (3)
C15—C14—H14B	109.2	C52—C51—P2	110.7 (2)
C13—C14—H14B	109.2	C56—C51—P2	118.0 (2)
H14A—C14—H14B	107.9	C52—C51—H51	105.5
C14—C15—C16	112.4 (4)	C56—C51—H51	105.5
C14—C15—H15A	109.1	P2—C51—H51	105.5
C16—C15—H15A	109.1	C53—C52—C51	112.4 (3)
C14—C15—H15B	109.1	C53—C52—H52A	109.1
C16—C15—H15B	109.1	C51—C52—H52A	109.1
H15A—C15—H15B	107.8	C53—C52—H52B	109.1
C11—C16—C15	111.6 (3)	C51—C52—H52B	109.1
C11—C16—H16A	109.3	H52A—C52—H52B	107.9
C15—C16—H16A	109.3	C52—C53—C54	112.5 (4)
C11—C16—H16B	109.3	C52—C53—H53A	109.1
C15—C16—H16B	109.3	C54—C53—H53A	109.1
H16A—C16—H16B	108	C52—C53—H53B	109.1
C22—C21—C26	109.0 (3)	C54—C53—H53B	109.1
C22—C21—P1	110.0 (2)	H53A—C53—H53B	107.8
C26—C21—P1	112.2 (2)	C55—C54—C53	110.9 (3)
C22—C21—H21	108.5	C55—C54—H54A	109.5
C26—C21—H21	108.5	C53—C54—H54A	109.5
P1—C21—H21	108.5	C55—C54—H54B	109.5
C23—C22—C21	111.9 (3)	C53—C54—H54B	109.5
C23—C22—H22A	109.2	H54A—C54—H54B	108
C21—C22—H22A	109.2	C54—C55—C56	111.8 (3)
C23—C22—H22B	109.2	C54—C55—H55A	109.2
C21—C22—H22B	109.2	C56—C55—H55A	109.2
H22A—C22—H22B	107.9	C54—C55—H55B	109.2
C22—C23—C24	110.5 (3)	C56—C55—H55B	109.2
C22—C23—H23A	109.5	H55A—C55—H55B	107.9
C24—C23—H23A	109.5	C55—C56—C51	111.2 (3)
C22—C23—H23B	109.5	C55—C56—H56A	109.4
C24—C23—H23B	109.5	C51—C56—H56A	109.4
H23A—C23—H23B	108.1	C55—C56—H56B	109.4
C25—C24—C23	111.1 (3)	C51—C56—H56B	109.4
C25—C24—H24A	109.4	H56A—C56—H56B	108
C23—C24—H24A	109.4	C62—C61—C66	117.5 (3)
C25—C24—H24B	109.4	C62—C61—P2	119.2 (3)
C23—C24—H24B	109.4	C66—C61—P2	123.2 (3)
H24A—C24—H24B	108	C63—C62—C61	121.3 (4)
C24—C25—C26	112.0 (4)	C63—C62—H62	119.4
C24—C25—H25A	109.2	C61—C62—H62	119.4
C26—C25—H25A	109.2	C64—C63—C62	120.3 (4)
C24—C25—H25B	109.2	C64—C63—H63	119.9
C26—C25—H25B	109.2	C62—C63—H63	119.9
H25A—C25—H25B	107.9	C65—C64—C63	119.7 (4)
C25—C26—C21	109.7 (3)	C65—C64—H64	120.1
C25—C26—H26A	109.7	C63—C64—H64	120.1
C21—C26—H26A	109.7	C64—C65—C66	120.7 (4)
C25—C26—H26B	109.7	C64—C65—H65	119.7
C21—C26—H26B	109.7	C66—C65—H65	119.7
H26A—C26—H26B	108.2	C61—C66—C65	120.5 (4)
C36—C31—C32	117.8 (3)	C61—C66—H66	119.8
C36—C31—P1	117.2 (2)	C65—C66—H66	119.8
C32—C31—P1	124.9 (3)	C75—N—C71	114.5 (8)
C33—C32—C31	120.5 (4)	N—C71—C72	122.1 (9)
C33—C32—H32	119.7	N—C71—H71	119
C31—C32—H32	119.7	C72—C71—H71	119
C34—C33—C32	120.5 (3)	C73—C72—C71	117.8 (10)
C34—C33—H33	119.7	C73—C72—H72	121.1
C32—C33—H33	119.7	C71—C72—H72	121.1
C35—C34—C33	119.3 (4)	C74—C73—C72	121.1 (10)
C35—C34—H34	120.4	C74—C73—H73	119.4
C33—C34—H34	120.4	C72—C73—H73	119.4
C34—C35—C36	120.6 (4)	C73—C74—C75	119.3 (10)
C34—C35—H35	119.7	C73—C74—H74	120.4
C36—C35—H35	119.7	C75—C74—H74	120.4
C35—C36—C31	121.0 (3)	C74—C75—N	125.0 (9)
C35—C36—H36	119.5	C74—C75—H75	117.5
C31—C36—H36	119.5	N—C75—H75	117.5
C46—C41—C42	110.2 (3)		
			
P2—Ag—P1—C31	−54.39 (13)	C34—C35—C36—C31	2.5 (7)
I—Ag—P1—C31	134.97 (12)	C32—C31—C36—C35	−1.4 (6)
P2—Ag—P1—C21	67.00 (12)	P1—C31—C36—C35	−176.7 (3)
I—Ag—P1—C21	−103.64 (11)	C61—P2—C41—C46	−66.3 (3)
P2—Ag—P1—C11	−175.78 (11)	C51—P2—C41—C46	−176.6 (2)
I—Ag—P1—C11	13.58 (12)	Ag—P2—C41—C46	62.6 (2)
P1—Ag—P2—C61	177.17 (12)	C61—P2—C41—C42	171.3 (3)
I—Ag—P2—C61	−13.59 (13)	C51—P2—C41—C42	61.1 (3)
P1—Ag—P2—C51	−63.97 (12)	Ag—P2—C41—C42	−59.7 (3)
I—Ag—P2—C51	105.26 (11)	C46—C41—C42—C43	54.0 (4)
P1—Ag—P2—C41	54.26 (12)	P2—C41—C42—C43	176.1 (3)
I—Ag—P2—C41	−136.51 (11)	C41—C42—C43—C44	−55.5 (5)
C31—P1—C11—C16	63.3 (3)	C42—C43—C44—C45	56.8 (5)
C21—P1—C11—C16	−47.6 (3)	C43—C44—C45—C46	−56.5 (5)
Ag—P1—C11—C16	−168.9 (2)	C42—C41—C46—C45	−55.2 (4)
C31—P1—C11—C12	−65.9 (3)	P2—C41—C46—C45	−178.2 (3)
C21—P1—C11—C12	−176.8 (2)	C44—C45—C46—C41	56.8 (5)
Ag—P1—C11—C12	61.9 (2)	C61—P2—C51—C52	70.1 (3)
C16—C11—C12—C13	53.9 (4)	C41—P2—C51—C52	179.9 (2)
P1—C11—C12—C13	−175.0 (2)	Ag—P2—C51—C52	−55.4 (3)
C11—C12—C13—C14	−53.5 (5)	C61—P2—C51—C56	−58.7 (3)
C12—C13—C14—C15	53.4 (5)	C41—P2—C51—C56	51.1 (3)
C13—C14—C15—C16	−53.6 (5)	Ag—P2—C51—C56	175.8 (2)
C12—C11—C16—C15	−53.8 (4)	C56—C51—C52—C53	−53.4 (4)
P1—C11—C16—C15	176.5 (3)	P2—C51—C52—C53	174.0 (3)
C14—C15—C16—C11	54.1 (5)	C51—C52—C53—C54	53.2 (5)
C31—P1—C21—C22	−175.3 (2)	C52—C53—C54—C55	−53.4 (6)
C11—P1—C21—C22	−66.0 (2)	C53—C54—C55—C56	55.2 (5)
Ag—P1—C21—C22	57.7 (2)	C54—C55—C56—C51	−56.6 (5)
C31—P1—C21—C26	63.2 (3)	C52—C51—C56—C55	54.9 (4)
C11—P1—C21—C26	172.5 (2)	P2—C51—C56—C55	−176.3 (3)
Ag—P1—C21—C26	−63.8 (3)	C51—P2—C61—C62	−110.0 (3)
C26—C21—C22—C23	−58.3 (4)	C41—P2—C61—C62	139.9 (3)
P1—C21—C22—C23	178.2 (2)	Ag—P2—C61—C62	11.3 (3)
C21—C22—C23—C24	56.0 (4)	C51—P2—C61—C66	69.5 (3)
C22—C23—C24—C25	−53.9 (5)	C41—P2—C61—C66	−40.6 (3)
C23—C24—C25—C26	56.1 (5)	Ag—P2—C61—C66	−169.2 (3)
C24—C25—C26—C21	−58.5 (5)	C66—C61—C62—C63	0.0 (6)
C22—C21—C26—C25	58.4 (4)	P2—C61—C62—C63	179.6 (4)
P1—C21—C26—C25	−179.6 (3)	C61—C62—C63—C64	0.3 (8)
C21—P1—C31—C36	−159.5 (3)	C62—C63—C64—C65	−0.1 (8)
C11—P1—C31—C36	91.4 (3)	C63—C64—C65—C66	−0.6 (8)
Ag—P1—C31—C36	−35.5 (3)	C62—C61—C66—C65	−0.7 (6)
C21—P1—C31—C32	25.6 (3)	P2—C61—C66—C65	179.8 (3)
C11—P1—C31—C32	−83.5 (3)	C64—C65—C66—C61	0.9 (7)
Ag—P1—C31—C32	149.5 (3)	C75—N—C71—C72	4.4 (13)
C36—C31—C32—C33	2.8 (5)	N—C71—C72—C73	−6.7 (15)
P1—C31—C32—C33	177.7 (3)	C71—C72—C73—C74	4.5 (17)
C31—C32—C33—C34	−5.4 (6)	C72—C73—C74—C75	−0.2 (17)
C32—C33—C34—C35	6.5 (6)	C73—C74—C75—N	−2.3 (16)
C33—C34—C35—C36	−5.0 (7)	C71—N—C75—C74	0.1 (14)

### Hydrogen-bond geometry (Å, °)

**Table:**

*D*—H···*A*	*D*—H	H···*A*	*D*···*A*	*D*—H···*A*
C66—H66···N^i^	0.93	2.72	3.538 (4)	147

Symmetry codes: (i) −*x*+1, −*y*+1, −*z*+1.
